# A case of sepsis‐induced cardiomyopathy successfully treated with venoarterial extracorporeal membrane oxygenation

**DOI:** 10.1002/iju5.12540

**Published:** 2022-10-05

**Authors:** Keigo Sato, Akihiro Naito, Taichi Shiratori, Masahiro Yamamoto, Kenichi Shimane, Manabu Mikami, Mariko Senda, Haruki Kume, Motofumi Suzuki

**Affiliations:** ^1^ Department of Urology Tokyo Metropolitan Bokutoh Hospital Tokyo Japan; ^2^ Department of Urology Chiba Tokushukai Hospital Funabashi Japan; ^3^ Department of Rheumatology Tokyo Metropolitan Bokutoh Hospital Tokyo Japan; ^4^ Tertiary Emergency Medical Center Tokyo Metropolitan Bokutoh Hospital Tokyo Japan; ^5^ Center for Antarctic Programs National Institute of Polar Research Tachikawa Japan; ^6^ Department of Anesthesiology Tokyo Metropolitan Bokutoh Hospital Tokyo Japan; ^7^ Department of Urology, Graduate School of Medicine The University of Tokyo Tokyo Japan

**Keywords:** cardiomyopathy, echocardiography, left ventricular ejection fraction, sepsis, urolithiasis, venoarterial extracorporeal membrane oxygenation

## Abstract

**Introduction:**

Sepsis with concomitant acute pyelonephritis, secondary to urolithiasis, is common. We report a case of sepsis‐induced cardiomyopathy with acute pyelonephritis, successfully managed with venoarterial extracorporeal membrane oxygenation.

**Case presentation:**

A 64‐year‐old woman presented with fever and disturbed consciousness. Abdominal computed tomography revealed right hydronephrosis with ipsilateral ureteral stone. Despite ureteral stent placement and antibiotic treatment, her hemodynamics worsened. She was diagnosed with sepsis‐induced cardiomyopathy and underwent venoarterial extracorporeal membrane oxygenation. Her hemodynamics improved rapidly; venoarterial extracorporeal membrane oxygenation was withdrawn on postoperative day‐3. She was discharged from our hospital after sufficient antibiotic treatment.

**Conclusion:**

Venoarterial extracorporeal membrane oxygenation may be initiated in patients with sepsis‐induced cardiomyopathy. Evaluation of left ventricular ejection fraction via echocardiography is important to determine the indication for venoarterial extracorporeal membrane oxygenation.

**Abbreviations & Acronyms**
ESBL: extended‐spectrum beta‐lactamase
ICU: intensive care unit
LVEF: left ventricular ejection fraction
POD: postoperative day
SIC: sepsis‐induced cardiomyopathy
UTI: urinary tract infection
VA ECMO: venoarterial extracorporeal membrane oxygenation

**Keynote message**

Venoarterial extracorporeal membrane oxygenation may be used for patients with sepsis‐induced cardiomyopathy due to urolithiasis. Echocardiographic evaluation of left ventricular ejection fraction should be considered to determine the indication for venoarterial extracorporeal membrane oxygenation if the patient's hemodynamics remains unstable or worsens despite optimized surgical intervention, fluid resuscitation, antibiotics, and high‐dose inotropes and vasopressors. Flexible and immediate responses by medical staff are required in managing patients with sepsis‐induced cardiomyopathy.

## Introduction

Acute pyelonephritis with upper urinary tract stones is a common emergent condition that may develop into urosepsis, requiring surgical interventions and admission to the ICU; however, it can usually be managed by drainage with a ureteral stent or percutaneous nephrostomy, infection control with antibiotics, and respiratory and circulatory support. Urosepsis can rarely lead to SIC, an acute myocardial impairment with high mortality. VA ECMO is utilized as a mechanical circulatory support for patients with refractory sepsis, including SIC. In the present report, we have reviewed the clinical course of the extremely rare adult female case of SIC with acute pyelonephritis, successfully managed with VA ECMO.

## Case presentation

In February 2021, a 64‐year‐old woman was brought to our emergency room due to disturbed consciousness. Her Charlson Comorbidity Index^1^ score was 2 (rheumatoid arthritis and bronchial asthma). One day before admission, the patient was febrile, experienced general fatigue, and vomited three times. On admission, her level of consciousness was I–2 on the Japan Coma Scale.^2^ Her blood pressure was 91/60 mmHg and respiratory rate was 22 breaths per minute, giving her a quick Sequential Organ Failure Assessment Score of 3.^3^

**Fig. 1:**
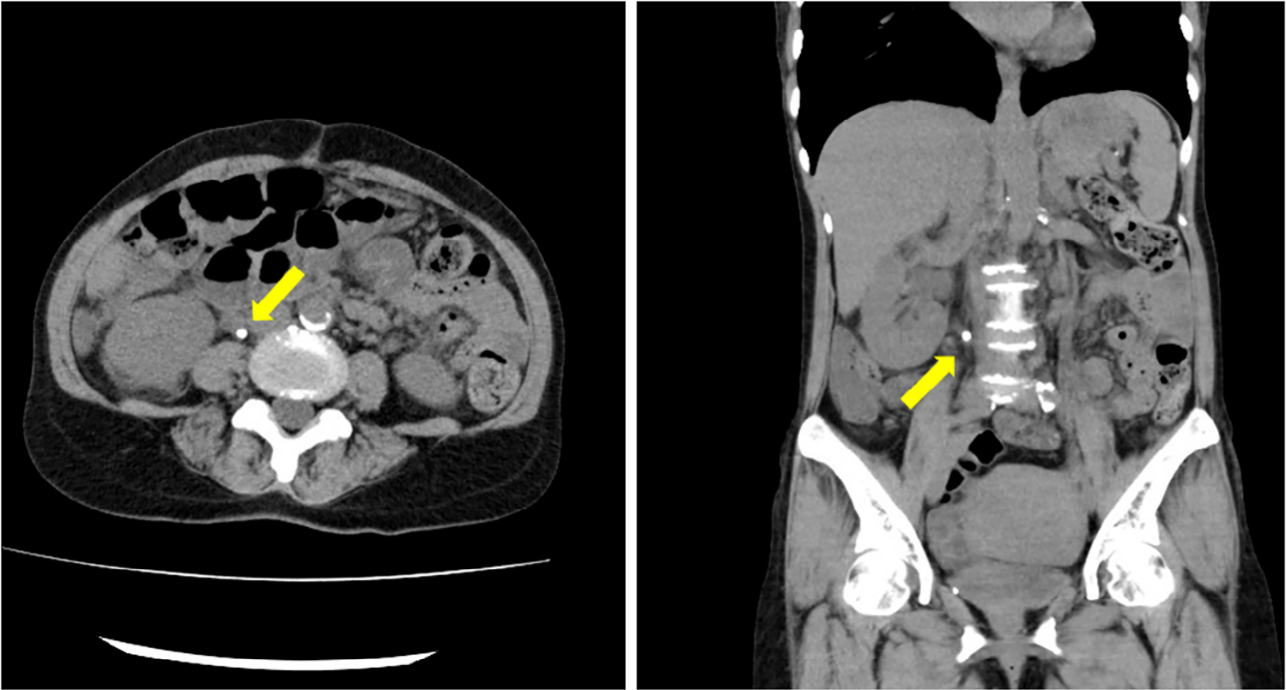
Images of abdominal computed tomography. The left figure is axial, and the right figure is the coronal view. Yellow arrowheads indicate a right ureter stone. The right ureter stone is located at the ureteropelvic junction. Right hydronephrosis is also shown in the coronal view.

Abdominal computed tomography revealed right hydronephrosis due to a 6‐mm upper ureter stone (Fig. 1). She was diagnosed with urosepsis due to right acute pyelonephritis. Preoperatively, her serum lactate level was 5.8 mmol/L. We indwelled a 6‐Fr 24‐cm silicon ureteral stent into her right ureter. Postoperatively, her systolic blood pressure was 110 mmHg with the support of a continuous injection of noradrenaline at 0.20 μg/kg/min. Transthoracic echocardiography revealed a LVEF of 60%. Since her hemodynamics remained unstable, we transferred her to the coronary care unit and initiated systemic management. Three hours after surgery, her blood pressure decreased to 49/42 mmHg despite intensive treatment with noradrenaline, vasopressin, and dobutamine. Transthoracic echocardiography revealed a decreased LVEF of 20% with severe diffuse hypokinesis. No asynergy was recorded. Four hours postoperatively, she was diagnosed with SIC, and VA ECMO was initiated immediately. She was administered meropenem to protect against bacterial infections, including ESBL‐producing strains. Her condition improved gradually. VA ECMO and vasopressor agents were discontinued on POD‐3, and she was extubated on POD‐4. Her LVEF recovered to 71%, without asynergy.

**Fig. 2:**
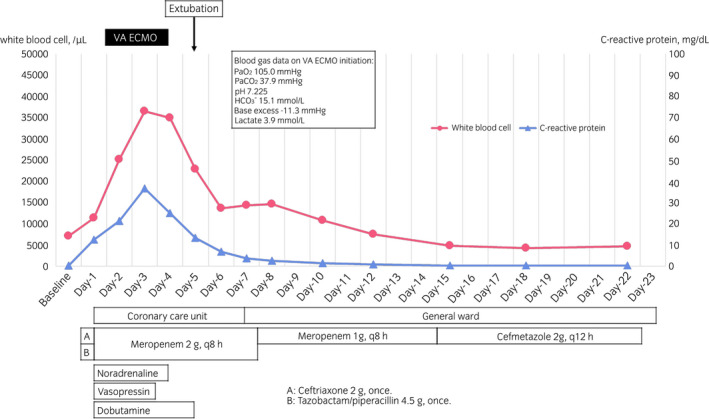
Time course of white blood cell count and C‐reactive protein. The *X*‐axis indicates the days of the hospital stay.

On POD‐3, ESBL‐producing *Escherichia coli* was detected in both urine and peripheral blood cultures. On POD‐7, she was transferred to the general ward, and rehabilitation therapy was initiated. She was discharged from our hospital on POD‐23 (Fig. 2). Since her ureter stone size was 6 mm in diameter, we expected that it would be excreted naturally. However, the stone position remained unchanged for 6 months. Subsequently, we surgically removed the ureter stone using a basket catheter without lithotripsy in August 2021. The stone was composed of 57% calcium oxalate and 43% calcium phosphate. Fourteen months later, she recovered well without any recurrence of urinary stones or symptomatic UTI.

## Discussion

Cardiovascular collapse in patients undergoing septic shock includes increased vasodilation and SIC.^4^ Habimana *et al*. described the pathophysiology of SIC as involving impaired myocardial circulation, direct myocardial depression, and mitochondrial dysfunction. The mechanisms underlying direct myocardial depression include downregulation of β‐adrenoceptors and several myocardial suppressants. Apoptosis of cardiomyocytes is among the leading causes of myocardial depression and sepsis‐induced multiple organ dysfunction.^5^ Although SIC is a form of transient cardiac dysfunction, the exact diagnostic criteria for this condition remain unclear.^6^ The prevalence of SIC is 30–50% in patients with sepsis having an ejection fraction <45%.^7^, ^8^ Among the patients with sepsis in the ICU, the risk of mortality for patients with SIC was higher than that for patients without SIC (42.1% *vs* 17.6%; *P* = 0.008). SIC was also associated with an increased risk of all‐cause in‐hospital mortality (odds ratio, 4.46; 95% confidence interval, 1.15–18.69; *P* = 0.031).^9^

VA ECMO is utilized as a mechanical circulatory support, mainly for patients with medical (acute myocardial infarction, fulminant myocarditis, intoxication with cardiotoxic drugs, end‐stage dilated or ischemic cardiomyopathy, hypothermia with refractory cardiocirculatory instability, and massive pulmonary embolism), and postsurgical (including post‐transplantation) acute cardiogenic shock.^10^ Despite the emerging use of VA ECMO for the treatment of SIC,^10^ its use in adults with SIC is not as effective as in children with SIC.^11^ The hemodynamic pattern of septic shock differs markedly across age groups: newborn infants typically present with pulmonary hypertension and right heart failure, young children with left heart failure, and adolescents and adults with distributive shock.^12^ Recently, Ling *et al*. reported that the pre‐ECMO LVEF was significantly associated with mortality. The survival rates among patients with LVEF >35%, LVEF between 20% and 35%, and LVEF <20% were 32.1%, 42.3%, and 62.0%, respectively.^11^ This means that a higher pre‐ECMO LVEF results in lower survival. In septic patients with preserved cardiac function, VA ECMO may be contraindicated due to decreasing cardiac output.^11^, ^13^ According to the Extracorporeal Life Support Organization interim guidelines for adult cardiac patients, VA ECMO should be initiated within 6 h in patients with cardiogenic shock refractory to conventional pharmacological and fluid therapy and in patients with reversible cardiocirculatory assistance.^10^ As Bréchot *et al*. suggested, VA ECMO could decrease vasopressor dose rapidly and restore adequate perfusion of vital organs.^14^

To the best of our knowledge, this is the second case report regarding SIC caused by acute pyelonephritis with urolithiasis successfully managed by VA ECMO. In 2020, Zhu *et al*. reported the first case of refractory septic shock with SIC after treating a ureter stone by holmium laser lithotripsy.^15^ The female patient aged 67 years suffered from heart, lung, and kidney failure after 48 h of lithotripsy and was transferred to the ICU for intensive treatment including VA ECMO. The patient required amputation of both upper and lower extremities and intermittent hemodialysis and survived despite a poor clinical course. Since purulent urine was observed during the operation, we thought that such serious complications could have been avoided if a ureteral stent had been indwelled first to manage UTI and secondary lithotripsy had been performed. Timely initiation of VA ECMO in appropriate patients might also be important for a good outcome.

## Conclusion

We should consider echocardiography to determine the indication for VA ECMO if the patient's hemodynamics remain unstable or worsen despite optimized surgical intervention, fluid resuscitation, antibiotics, and high‐dose inotropes and vasopressors. VA ECMO may be a key treatment option for patients experiencing SIC due to acute pyelonephritis with urolithiasis.

## Author contributions

Keigo Sato: Visualization; writing – original draft; writing – review and editing. Akihiro Naito: Visualization; writing – review and editing. Taichi Shiratori: Writing – review and editing. Masahiro Yamamoto: Writing – review and editing. Kenichi Shimane: Writing – review and editing. Manabu Mikami: Writing – review and editing. Mariko Senda: Writing – review and editing. Haruki Kume: Writing – review and editing. Motofumi Suzuki: Conceptualization; project administration; supervision; writing – original draft; writing – review and editing.

## Conflict of interest

The authors declare no conflict of interest.

## Approval of the research protocol by an Institutional Reviewer Board

This study was approved by the Institutional Reviewer Board of the Tokyo Metropolitan Bokutoh Hospital (approved No. 02‐142‐02).

## Informed consent

Written informed consent was obtained from the patient.

## Registry and the Registration No. of the study/trial

Not applicable.
